# Supplementary material for: A scoping review of substance use brief interventions in Africa
Source: PLOS Glob Public Health. 2024 Oct 24;4(10):e0003340. doi: 10.1371/journal.pgph.0003340 (PMC11501030; doi:10.1371/journal.pgph.0003340)
Supplement: S3 File — (PDF) [file pgph.0003340.s004.pdf]

EBSCOhost

Tuesday, October 31, 2023 9:40:45 PM

#	Query	Limiters/Expanders	Last Run Via	Results
S1	(brief intervention OR brief treatment OR Screening and Brief intervention OR screening brief intervention and referral to treatment OR SBIRT OR motivational interviewing OR brief counselling) AND (Substance use OR substance use disorder OR drug use OR alcohol OR harmful drinking OR tobacco OR smoking OR Cigarette OR khat OR cannabis OR opioid OR heroin OR stimulant OR methamphetamine OR illicit drugs OR addiction treatment) AND (Africa OR Algeria OR Angola OR Benin OR Botswana OR Burkina Faso OR Burundi OR Cameroon OR Cape Verde OR Central African Republic OR Chad OR Comoros OR Democratic Republic of the Congo OR Republic of the Congo OR Djibouti OR Egypt OR Equatorial Guinea OR Eritrea OR Ethiopia OR Gabon OR Gambia OR Ghana OR Guinea OR Guinea-Bissau OR Ivory Coast OR Kenya OR Lesotho OR Liberia OR Libya OR Madagascar OR Malawi OR Mali OR Mauritania OR Mauritius OR Morocco OR Mozambique OR Namibia OR Niger OR Nigeria OR Rwanda OR Sao Tome and Principe OR Senegal OR Seychelles OR Sierra Leone OR Somalia OR South Africa OR South Sudan OR Sudan OR Swaziland OR Tanzania OR Togo OR Tunisia OR Uganda OR Zambia OR Zimbabwe)	Expanders - Apply equivalent subjects<br>Search modes - Boolean/Phrase	Interface - EBSCOhost Research Databases<br>Search Screen - Advanced Search<br>Database - CINAHL with Full Text	98
