# Supplementary material for: A scoping review of substance use brief interventions in Africa
Source: PLOS Glob Public Health. 2024 Oct 24;4(10):e0003340. doi: 10.1371/journal.pgph.0003340 (PMC11501030; doi:10.1371/journal.pgph.0003340)
Supplement: S5 File — (PDF) [file pgph.0003340.s006.pdf]

# Advanced Search

Search | Search manager | Medical terms (MeSH) | PICO search

Save search | View saved searches | ? Search help

Did you know you can now select fields from Search manager using the S button (next to the search box)?
Search manager lets you add unlimited search lines, view results per line and access the MeSH browser using the new MeSH button.

| | | |
|---|---|---|
| All Text | | brief intervention OR brief treatment OR Screening and Brief intervention OR screening brief intervention and referral to treatment OR SBIRT OR motivational interviewing OR brief |
| AND | Title Abstract Keyword | Substance use OR substance use disorder OR drug use OR alcohol OR harmful drinking OR tobacco OR smoking OR Cigarette OR khat OR cannabis OR opioid OR h |
| AND | Title Abstract Keyword | Africa OR Algeria OR Angola OR Benin OR Botswana OR Burkina Faso OR Burundi OR Cameroon OR Cape Verde OR Central African Republic OR Chad OR Comoros |

(Word variations have been searched)

Search limits | Send to search manager | Run search

Clear all

## Filter your results

### Year

**Year first published**

- 2023 ... 23
- 2022 ... 26
- 2021 ... 33
- 2020 ... 23
- 2019 ... 41

**Custom Range:**
yyyy to yyyy
Apply | Clear

### Date

**Date added to CENTRAL trials database**

- The last 3 months ... 5
- The last 6 months ... 16
- The last 9 months ... 24
- The last year ... 26
- The last 2 years ... 76

**Custom Range:**
dd/mm/yyyy to dd/mm/yyyy
Apply | Clear

### Source

- PubMed ... 146
- Embase ... 123
- CT.gov ... 55
- CINAHL ... 19
- ICTRP ... 12

### Language

- English ... 274
- French ... 1

Cochrane Reviews 74 | Cochrane Protocols 0 | Trials 275 | Editorials 0 | Special Collections 0 | Clinical Answers 0 | More

For COVID-19 related studies, please also see the **Cochrane COVID-19 Study Register**

**275** Trials matching **brief intervention OR brief treatment OR Screening and Brief intervention OR screening brief intervention and referral to treatment OR SBIRT OR motivational interviewing OR brief counselling in All Text AND Substance use OR substance use disorder OR drug use OR alcohol OR harmful drinking OR tobacco OR smoking OR Cigarette OR khat OR cannabis OR opioid OR heroin OR stimulant OR methamphetamine OR illicit drugs OR addiction treatment in Title Abstract Keyword AND Africa OR Algeria OR Angola OR Benin OR Botswana OR Burkina Faso OR Burundi OR Cameroon OR Cape Verde OR Central African Republic OR Chad OR Comoros OR Democratic Republic of the Congo OR Republic of the Congo OR Djibouti OR Egypt OR Equatorial Guinea OR Eritrea OR Ethiopia OR Gabon OR Gambia OR Ghana OR Guinea OR Guinea-Bissau OR Ivory Coast OR Kenya OR Lesotho OR Liberia OR Libya OR Madagascar OR Malawi OR Mali OR Mauritania OR Mauritius OR Morocco OR Mozambique OR Namibia OR Niger OR Nigeria OR Rwanda OR Sao Tome and Principe OR Senegal OR Seychelles OR Sierra Leone OR Somalia OR South Africa OR South Sudan OR Sudan OR Swaziland OR Tanzania OR Togo OR Tunisia OR Uganda OR Zambia OR Zimbabwe in Title Abstract Keyword - (Word variations have been searched)**

Cochrane Central Register of Controlled Trials
Issue 10 of 12, October 2023

Deselect all (275) | Export selected citation(s)

Order by Relevancy | Results per page 25

1. **Qualitative perceptions of dapivirine vaginal ring adherence and drug level feedback following an open-label extension trial**
K Naidoo, LE Mansoor, AW Katz, M Garcia, D Kemigisha, NS Morar, CC Zimba, M Chitukuta, K Reddy, L Soto-Torres, S Naidoo, ET Montgomery
Journal of acquired immune deficiency syndromes (1999), **2020** | added to CENTRAL: 31 January 2021 | 2021 Issue 01
PubMed | Embase

2. **“striving and hoping”-women's motivations to join an HIV prevention clinical trial: narratives from the voice-D study**
A Katz, K Woeber, P Musara, J Etima, B Mensch, A Van Der Straten
AIDS research and human retroviruses, **2016**, 32, 150 | added to CENTRAL: 31 October 2018 | 2018 Issue 10
Embase

3. **Effectiveness of an Evidence-based Stepped Care System for Alcohol and Other Drug Use Problems Among Congolese Refugees in Zambia**
NCT05471921
https://clinicaltrials.gov/show/NCT05471921, **2022** | added to CENTRAL: 31 August 2022 | 2022 Issue 8
CT.gov

4. **Trial of a Multi-pronged Intervention to Address Prevention of Violence in Zambia**
NCT02790827
https://clinicaltrials.gov/show/NCT02790827, **2016** | added to CENTRAL: 31 May 2018 | 2018 Issue 5
CT.gov

5. **Development and integration of a novel cognitive-behavioral therapy-based alcohol brief intervention into public HIV clinics in Zambia**
S Skavenski, C Danielson, E Mupinde, C Mukunta, T Kanguya, A Sharma, M Vinikoor, J Kane, L Murray
Alcoholism: clinical and experimental research, **2021**, 45(SUPPL 1), 78A | added to CENTRAL: 31 August 2021 | 2021 Issue 08
Embase

6. **Adapting and Evaluating a Tobacco Use Cessation Program for People Living With HIV in Uganda and Zambia**
NCT05487807
https://clinicaltrials.gov/show/NCT05487807, **2022** | added to CENTRAL: 31 August 2022 | 2022 Issue 8
CT.gov

7. **Substance abuse, treatment needs and access among female sex workers and non-sex workers in Pretoria, South Africa**
WM Wechsberg, LT Wu, WA Zule, CD Parry, FA Browne, WK Luseno, T Kline, A Gentry
Substance abuse treatment, prevention, and policy, **2009**, 4, 11 | added to CENTRAL: 31 March 2019 | 2019 Issue 3
PubMed | Embase

8. **Alcohol, cannabis, and methamphetamine use and other risk behaviours among Black and Coloured South African women: a small randomized trial in the Western Cape**
WM Wechsberg, WK Luseno, RS Karg, S Young, N Rodman, B Myers, CD Parry
International journal on drug policy, **2008**, 19(2), 130-139 | added to CENTRAL: 31 July 2008 | 2008 Issue 3
PubMed

9. **Common Elements Treatment Approach HIV Alcohol Reduction Trial in Zambia**
NCT05121064
https://clinicaltrials.gov/show/NCT05121064, **2021** | added to CENTRAL: 30 November 2021 | 2021 Issue 11
CT.gov

10. **Zambia Common Elements Treatment Approach Pilot Study**
NCT03966885
https://clinicaltrials.gov/show/NCT03966885, **2019** | added to CENTRAL: 30 June 2019 | 2019 Issue 06
CT.gov

11. **Effectiveness of a psychological intervention delivered by general nurses for alcohol use disorders in people living with HIV in Zimbabwe: a cluster randomized controlled trial**
M Madhombiro, M Kidd, B Dube, M Dube, W Mutsvuke, T Muronzie, DT Zhou, S Derveeuw, D Chibanda, A Chingono, S Rusakaniko, A Hutson, GD Morse, MA Abas, S Seedat
Journal of the International AIDS Society, **2020**, 23(12), e25641 | added to CENTRAL: 31 January 2021 | 2021 Issue 01
PubMed | Embase

12. **Brief counselling after home-based HIV counselling and testing strongly increases linkage to care: a cluster-randomized trial in Uganda**
E Ruzagira, H Grosskurth, A Kamali, K Baisley
Journal of the International AIDS Society, **2017**, 20(2) | added to CENTRAL: 31 March 2018 | 2018 Issue 3
PubMed | Embase

13. **The role of community health workers in improving HIV treatment outcomes in children: lessons learned from the ZENITH trial in Zimbabwe**
J Busza, E Dauya, T Bandason, V Simms, CD Chikwari, M Makamba, G Mchugh, S Munyati, P Chonzi, RA Ferrand
Health policy and planning, **2018**, 33(3), 328-334 | added to CENTRAL: 30 September 2018 | 2018 Issue 9
PubMed

14. **Results of a cluster-randomized trial of non-financial incentives to increase uptake of couples counselling and testing among clients attending PSI mobile HIV services in rural Zimbabwe**
EL Sibanda, M Tumushime, J Mufuka, S Gudukeya, S Napierala Mavedzenge, S Bautista-Arredondo, H Thirumurthy, S McCoy, N Padian, K Hatzold, A Copas, FM Cowan
Journal of the International AIDS Society, **2016**, 19, 62 | added to CENTRAL: 31 March 2019 | 2019 Issue 3
Embase

15. **Mobile Technology to Extend Clinic-based Counseling for HIV+s in Uganda**
NCT03928418
https://clinicaltrials.gov/show/NCT03928418, **2019** | added to CENTRAL: 31 May 2019 | 2019 Issue 05
CT.gov

16. **Efficacy of a Single, Brief Alcohol Reduction Intervention among Men and Women Living with HIV/AIDS and Using Alcohol in Kampala, Uganda: a Randomized Trial**
B Wandera, NM Tumwesigye, JI Nankabirwa, DK Mafigiri, RM Parkes-Ratanshi, S Kapiga, J Hahn, AK Sethi
Journal of the International Association of Providers of AIDS Care, **2017**, 16(3), 276-285 | added to CENTRAL: 30 June 2017 | 2017 Issue 6
PubMed | Embase

17. **Integration of Common Elements Treatment Approach (CETA) into public sector HIV clinics for unhealthy alcohol use in urban Zambia: qualitative evaluation on acceptability and feasibility**
ME Lasater, T Kanguya, J Chipungu, JC Kane, S Skavenski, LK Murray, M Vinikoor, A Sharma
SSM - mental health, **2023**, 3 | added to CENTRAL: 31 May 2023 | 2023 Issue 5
Embase

18. **Intervention for alcohol use disorders at an HIV care clinic in Harare: a pilot and feasibility study**
M Madhombiro, B Dube, M Dube, M Zunza, D Chibanda, S Rusakaniko, S Seedat
Addiction science & clinical practice, **2019**, 14(1), 16 | added to CENTRAL: 30 June 2019 | 2019 Issue 06
PubMed | Embase

19. **Alcohol-focused and transdiagnostic treatments for unhealthy alcohol use among adults with HIV in Zambia: a 3-arm randomized controlled trial**
MJ Vinikoor, A Sharma, LK Murray, CJ Figge, S Bosomprah, C Chitambi, R Paul, T Kanguya, S Sivile, V Nghiem, K Cropsey, JC Kane
Contemporary clinical trials, **2023**, 127, 107116 | added to CENTRAL: 31 March 2023 | 2023 Issue 3
PubMed | Embase

20. **Common Elements Treatment Approach (CETA) for unhealthy alcohol use among persons with HIV in Zambia: study protocol of the ZCAP randomized controlled trial**
JC Kane, A Sharma, LK Murray, G Chander, T Kanguya, ME Lasater, S Skavenski, R Paul, J Mayeya, C Kmett Danielson, J Chipungu, C Chitambi, MJ Vinikoor
Addictive behaviors reports, **2020**, 12 | added to CENTRAL: 30 June 2020 | 2020 Issue 06
Embase

21. **Randomized trial of common elements treatment approach for unhealthy alcohol use and psychiatric comorbidity among persons living with HIV in Zambia**
JC Kane, A Sharma, LK Murray, G Chander, T Kanguya, S Skavenski, C Chitambi, ME Lasater, R Paul, J Mayeya, K Cropsey, S Bosomprah, CK Danielson, J Chipungu, MJ Vinikoor
Alcoholism: clinical and experimental research, **2021**, 45(SUPPL 1), 78A | added to CENTRAL: 31 August 2021 | 2021 Issue 08
Embase

22. **Decreases in self-reported alcohol consumption following HIV counseling and testing at Mulago Hospital, Kampala, Uganda**
JA Hahn, R Fatch, RK Wanyenze, S Baveewo, MR Kamya, DR Bangsberg, TJ Coates
BMC infectious diseases, **2014**, 14(1) | added to CENTRAL: 28 February 2015 | 2015 Issue 2
Embase

23. **Brief Report: ritonavir Concentrations in Hair Predict Virologic Outcomes in HIV-Infected Adolescents With Virologic Failure on Atazanavir-Based or Ritonavir-Based Second-Line Treatment**
TD Chawana, CFB Nhachi, K Nathoo, B Ngara, H Okochi, A Louie, K Kuncze, D Katzenstein, J Metcalfe, M Gandhi
Journal of acquired immune deficiency syndromes (1999), **2021**, 88(2), 181-185 | added to CENTRAL: 31 July 2021 | 2021 Issue 07
PubMed | Embase

24. **Effectiveness of nurse-practitioner-delivered brief motivational intervention for young adult alcohol and drug use in primary care in South Africa: a randomized clinical trial**
JR Mertens, CL Ward, GF Bresick, T Broder, CM Weisner
Alcohol and alcoholism (Oxford, Oxfordshire), **2014**, 49(4), 430-438 | added to CENTRAL: 31 August 2014 | 2014 Issue 8
PubMed | Embase

25. **"Project YES! has given me a task to reach undetectable": qualitative findings from a peer mentoring program for youth living with HIV in Zambia**
KG Merrill, C Frimpong, VM Burke, EA Abrams, S Miti, JK Mwansa, JA Denison
PloS one, **2023**, 18(10), e0292719 | added to CENTRAL: 31 October 2023 | 2023 Issue 10
PubMed



Copyright © 2000 - 2023 by John Wiley & Sons, Inc. All Rights Reserved
Review our **Privacy Policy Cookie Policy Cookie Preferences**

WILEY
