# Supplementary material for: A scoping review of substance use brief interventions in Africa
Source: PLOS Glob Public Health. 2024 Oct 24;4(10):e0003340. doi: 10.1371/journal.pgph.0003340 (PMC11501030; doi:10.1371/journal.pgph.0003340)
Supplement: Attachment — Submitted filename: Response to reviewers.docx [file pgph.0003340.s007.docx]

**Response to reviewers**

We thank the reviewers for their constructive feedback. Please find below our responses. The line numbers indicated relate to the document with tracked changes. Corrections have also been highlighted in yellow.

**Reviewer #1 comments**

**A) Major comments**

**(1) 'For this study, BIs were defined as those delivered over 1-4 sessions whether the authors referred to them as brief or not'. Applying a numerical paradigm to define BIs based purely on number of sessions is problematic as the four sessions could be extensive and hence categorized under 'brief therapies' and not 'brief interventions'. I would suggest that the authors clarify in the paper that their inclusion criteria covers both brief interventions and brief therapies (provided they are less than 5 sessions). This is the piece that can be corrected as it is within the control of the authors.**

We agree with this comment. We settled on this definition of BIs following a preliminary search on PubMed that showed that there was significant heterogeneity in the way studies had defined substance use brief interventions. In fact, even though authorities like SAMHSA provide a definition of brief therapies vs BIs, they acknowledge that the distinction in practice is unclear. We have added a statement to this effect - line 130-134

In the discussion, we have called on authors to make a distinction between BIs and brief therapies since this will enable better synthesis of findings across studies- line 435

In the limitations section, we have added that our definition of BIs may have overlapped with that of brief therapies- line 475-477.

**2) Of the 974 abstracts that were screened almost 90% (n=872) were excluded. The reasons for exclusion include ineligibility or inability to retrieve the full text. It is unclear how many papers fall under the latter category, especially since the wording used indicates that most of those excluded were not retrievable ('We managed to retrieve 102 full texts'). It would be important to very clearly state how many were excluded because the papers were not retrievable as that has implications on the internal and external validity of your findings. This would not be correctable as this is beyond the control of the authors as they have already tried their best to retrieve the papers but were not successful**

860 articles were ineligible, and we did not find 12 full texts. We have clarified this in line 183 and on the PRISMA flow chart.

**B) Minor comments**

**(1) Abstract: There is nothing in the results about the effectiveness or feasibility of the interventions.**

We have added a summary of findings on line 35-36.

**(2) Please provide a rationale for conducting a scoping review and not a systematic review**

The goal of a scoping review is exploratory. We wanted to know the breadth of literature on BIs in Africa as well as explore the definition of BIs. This goal can be better achieved with a scoping review as compared with a systematic review which requires a more specific research question. We have added a statement and citation to this effect on line 85-86.

**(3) Please specify what study designs were eligible**

We have added this in line 127-128

**(4) While scoping review protocols are not accepted on Prospero one can register them on other portals such as OSF**

We acknowledge this comment. At the time of writing the manuscript, the authors were not aware of the existence of a repository. We thank the reviewers for this new knowledge.

**(5) In the tables that described the studies 'Study population, setting sample size; gender/age' are all lumped together. They should be separated in discrete columns.**

We would have liked to have them in separate columns, but the table could have been too wide given that we have other columns. We kindly request the reviewer and editor that the table is kept this way. We have separated out and organized the variables within that column for easy identification. See example below:

| Goldschmidt 2023 (42)  South Africa | Cross-sectional, survey | **Setting:** Healthcare facility, and community setting  **Study population**: adults (18 years or older)  **Sample size:** n= 54,187  **Gender:** Male 43%  **Age**: Median age 34; range (18-85 years) | **Screening:** AUDIT-C  **BI:** Brief advice on harms of alcohol use plus referral for high-risk users  **Duration:** 10-30min  **Substance:** alcohol  **Delivery:** lay counselors, In-person  **Intervention described as brief:** yes. |
| --- | --- | --- | --- |

**(6) Table 3: While the participants were the people who were trained, it is not clear what the target condition was i.e. who was the intervention delivered to. Please add a column to indicate that**

We have addressed this concern Table 4 line 347. See column labelled “description of brief intervention”.

**Reviewer #2:** 

**Title: The title is adequate and reflect the content of the manuscript.**

We thank the reviewer for this comment.

**Abstract: The abstract is generally well-written. However, it would be necessary to rework it to include a problem statement/introduction, as it currently begins directly with the objectives of the scoping review. The abstract could also be revised to be more concise, especially in the methods section.**

We have revised the background to include more context (line 21-23).

We have revised the methods section to make it more concise (line 24-31)

**Introduction: The introduction does not define what substance use refers to. At line 41, "inject drugs" is mentioned, and at line 42, "alcohol-attributable disease" is referenced. Several terms are used in the search strategy (Supporting File 1) such as alcohol, tobacco, cigarette, cannabis. However, these elements should be better specified.**

We have added a definition of substance use in the introduction section- Line 41

Definition of injecting drug use is provided in line 49.

line 42, "alcohol-attributable disease" is referenced – see line 54-59 for a definition

**Several terms are used in the search strategy (Supporting File 1) such as alcohol, tobacco, cigarette, cannabis. However, these elements should be better specified.**

This comment was unclear to the authors, but we have clarified in the manuscript that the names of substances we used in the search were obtained from the exploratory search as well as from our knowledge of the most commonly used substances in Africa. Line 121

**Line 54 should be more nuanced; while BIs can be used, this can be influenced by other factors such as the skills of healthcare professionals performing them, the contexts in which they are implemented, and other factors related to individuals using substances (e.g., social support, financial resources).**

We have added a statement to show that the effectiveness of BIs can be influenced by factors such as delivery setting, substances used and number of BI sessions- line 70-72.

**The objectives of the scoping review are relevant.**

Thank you for this comment

**Methods: At lines 79-80, it is mentioned that “an exploratory search was carried out to determine the extent of literature on substance use BIs in Africa.” However, no information is provided on how this exploratory search was conducted, the factors influencing the research question or objectives, or the selection of keywords used in the search strategy. Additionally, the databases used for this exploratory search are not specified. At lines 87-90, the search strategy is briefly explained.**

We have provided information on how the exploratory search was conducted and its outcome, and how it helped shape the objectives of the scoping review – line 98-112.

**It would be necessary to clarify why articles “published in English or translated to English” were included, considering that articles could be published in other languages.**

We only included articles published in English or had an English translation available because the authors were all English speaking and unable to interpret the findings of a paper published in another language. We have added it as a study limitation on line 477-480.

**Supporting File 1 presents the search strategy in PsychINFO. However, the strategy does not adhere to expected database search practices. Specifically, no Medical Subject Headings (MeSH) appear to have been included, and keywords do not use truncation (i.e., *) or quotation marks to include exact terms.**

We did not use Mesh terms. We however conducted an exploratory search and identified terms that related to our key words and included them individually in the search strategy.

We did not use truncation because they did not apply to our search terms.

**Additionally, several keywords or alternate terms are missing from each concept (e.g., e-cig, illicit substance, cannabinoids, genja, motivational intervention, risk reduction, harm reduction).**

We take note of these comments. The search terms we used were drawn from the exploratory search. Terms such as genja or risk-reduction, harm reduction, cannabinoids did not feature among the literature.

**It is mentioned that there is considerable heterogeneity in the definitions of BI. However, the specific definition adopted by the authors is not explicitly stated.**

The specific definition has been provided on line 130-134

**The process for abstract screening is generally well-explained. However, it is not mentioned whether pilot testing was conducted to standardize this process and enhance agreement among reviewers.**

Abstract screening was independently done by two authors (S.K and F.J) based on the predetermined eligibility criteria. Disagreements during this stage of the screening were resolved through discussion and consensus with a third author (E.T.) Line 143-145.

**The full-text review process is well-described, but it should be clarified who the third author was that was invited to resolve conflicts.**

We have added this to methods. Line 145

**Certain components of interventions are extracted (e.g., mode of delivery, number of sessions, duration). However, it would be necessary to extract all components of BIs to address the fifth objective of the scoping review, using the Template for Intervention Description and Replication (TIDieR).**

The authors believe that the current description of the intervention captures key elements in the TIDIER framework. The TIDIER template recommends that in describing an intervention, the following aspects be addressed: name of the intervention, theory and rationale for the intervention, physical or informational material provided to participants or used in intervention delivery; processes/procedures carried out in delivering the intervention; details of provider; mode of delivery; where the intervention was delivered; intervention dose, duration, intensity; adaptations made; modifications during the course of the study.

In our table, we have captured all components save for adaptations. We believe that a detailed description of those would be outside the scope of this scoping review.

**Results: Overall, this section is well-executed, but modifications should be made to enhance readability and deepen the extracted data.**

We thank the reviewers for this comment, we have made modifications to the tables. See comments below.

**The trend of article publications is presented. However, this element seems to have limited relevance for addressing the objectives of the scoping review as indicated in lines 59-62.**

We request to keep the trends as presenting it fits in with the goal of this scoping review which is to map the African BI literature and provide general characteristics of BI studies in Africa.

**A general description of the studies is provided at lines 155-158. However, it would be desirable to specify other more macroscopic results (e.g., design, targeted populations, type of BI, theories used) to offer a comprehensive overview of all 80 studies.**

In addition to the overall description of studies (line190-199), we have added a general description under each theme; see lines 202-215; line 260-272.

**It would be relevant to present sample sizes by type of study design (e.g., qualitative design X to X, cross-sectional study X to X, intervention studies X to X).**

In addition to the overall sample size ranges, (line198-199), we have added sample sizes under each theme; see lines 207-210; line 265 to 267; line 315-316

**Tables 1, 2, and 3 contain a large amount of pertinent information. It would be necessary to add a list of abbreviations at the bottom of these tables.**

This has been done after each table. See lines 229, 283, 323

**At line 191, it is mentioned that thirty-two studies explored multi-session BIs. However, this number does not seem to match the subsequent text.**

We have revised this on line 260. It is 30 studies and not 32.

**Table 2 has a similar format to Table 1. However, the studies should be rearranged alphabetically, as in Table 1. Alternatively, both tables should be organized by study design (e.g., quantitative, qualitative, mixed) and then alphabetically.**

We have revised both tables and arranged them alphabetically within each study design section.

**Discussion: The discussion is interesting, but it would be helpful to provide a reminder of the research question and highlight the most interesting results before delving into the discussion of different themes. It would also be valuable to address elements related to other BI outcomes (e.g., cost-effectiveness, knowledge, attitudes, practices on BIs).**

We have provided a reminder of the research question – line 413.

We have added discussion on the practice of SBI and economic evaluations line 449-462

**Research gaps: The section is interesting to provide avenues for reflection for researchers and stakeholders.**

Thank you for this comment

**Limitations: It is mentioned "the aim of this systematic review" on line 331. However, this should be changed to "scoping review." Since the search strategy was not comprehensive, it is not appropriate to indicate that this scoping review used a systematic approach.**

We have revised this. Line 473

**PRISMA Flowchart: An extra period is found after "Duplicates removed.". A number is missing after "Records after duplicates removed".**

We have removed the extra period.

**There is a discrepancy between the exclusion reasons stated in the PRISMA and the manuscript ("Intervention not meeting our definition of brief", "Intervention not targeting substance use").**

We have revised this on line 138 in exclusion criteria

**Additionally, the number of articles screened and included due to the hand search should be specified.**

We have clarified on line xxx that we did not get additional papers from the hand search. Line 119-120.
